# Chlorogenic Acids, Acting via Calcineurin, Are the Main Compounds in *Centella asiatica* Extracts That Mediate Resilience to Chronic Stress in *Drosophila melanogaster*

**DOI:** 10.3390/nu15184016

**Published:** 2023-09-16

**Authors:** Helen Holvoet, Dani M. Long, Liping Yang, Jaewoo Choi, Luke Marney, Burkhard Poeck, Claudia S. Maier, Amala Soumyanath, Doris Kretzschmar, Roland Strauss

**Affiliations:** 1 Institut für Entwicklungsbiologie und Neurobiologie, Johannes Gutenberg-Universität Mainz, 55128 Mainz, Germany; heholvoe@uni-mainz.de (H.H.);; 2 BENFRA Botanical Dietary Supplements Research Center, Oregon Health & Science University, Portland, OR 97239, USAliping.yang@oregonstate.edu (L.Y.); jaewoo.choi@oregonstate.edu (J.C.); soumyana@ohsu.edu (A.S.); 3 Oregon Institute of Occupational Health Sciences, Oregon Health & Science University, Portland, OR 97239, USA; 4 Department of Chemistry, Oregon State University, Corvallis, OR 97331, USA; 5 Linus Pauling Institute, Oregon State University, Corvallis, OR 97331, USA; 6 Department of Neurology, Oregon Health & Science University, Portland, OR 97239, USA

**Keywords:** Gotu Kola, caffeoylquinic acids, triterpenes, chronic stress, anhedonia

## Abstract

Common symptoms of depressive disorders include anhedonia, sleep problems, and reduced physical activity. Drugs used to treat depression mostly aim to increase serotonin signaling but these can have unwanted side effects. Depression has also been treated by traditional medicine using plants like *Centella asiatica* (CA) and this has been found to be well tolerated. However, very few controlled studies have addressed CA’s protective role in depression, nor have the active compounds or mechanisms that mediate this function been identified. To address this issue, we used *Drosophila melanogaster* to investigate whether CA can improve depression-associated symptoms like anhedonia and decreased climbing activity. We found that a water extract of CA provides resilience to stress induced phenotypes and that this effect is primarily due to mono-caffeoylquinic acids found in CA. Furthermore, we describe that the protective function of CA is due to a synergy between chlorogenic acid and one of its isomers also present in CA. However, increasing the concentration of chlorogenic acid can overcome the requirement for the second isomer. Lastly, we found that chlorogenic acid acts via calcineurin, a multifunctional phosphatase that can regulate synaptic transmission and plasticity and is also involved in neuronal maintenance.

## 1. Introduction

Depression currently affects about 350 million people worldwide, which is about 5% of the global population (https://www.who.int/newsroom/factsheets/detail/depression, (accessed on 1 June 2023)). This rate of depression is even higher in the United States with 8.4% (or 21 million) of adults experiencing at least one bout of depression in 2020 (https://www.nimh.nih.gov/health/statistics/major-depression, (accessed on 1 June 2023)). Although the symptoms vary, depression often manifests in feelings of helplessness, sadness, a loss of interest in activities the individual previously enjoyed, changes in appetite, and sleep problems. It can also result in thoughts about death and actual suicide [1], which is the second leading cause of death for 15–24 year olds (https://wmich.edu/suicideprevention/basic/facts, (accessed on 1 June 2023)), an age group that also shows a high rate of depression [2]. Another age group with higher rates of depression is the elderly, which again correlates with a high number of suicides that is approximately 50% higher than the average in the US [3,4,5]. 

Antidepressant drugs have become one of the most commonly prescribed medications. There are several classes of antidepressants, most of them modulators of serotonin levels with selective serotonin reuptake inhibitors (SSRIs) being the most frequently described type. Like other medications, their use is not without risk, causing side effects like insomnia, headache, dizziness, and blurred vision and their use has also been correlated to increased risk of bone fractures and mortality [6,7]. Especially because these medications are often taken over a long time, alternatives or supportive treatments with no or minimal adverse effects could benefit many patients. 

Traditional medicine has recently received growing interest as a means to promote human health and well-being, and the use of dietary supplements has increased dramatically, especially in the U.S. [8,9]. Botanical products have been used in Ayurvedic and Chinese medicine for thousands of years and recently health organizations like the World Health Organization (implementation of the WHO Traditional Medicine Strategy) or the National Institute of Health (NIH, Office of Dietary Supplements) have also shown interest in these treatment options. One of the plants used in traditional Asian medicine for brain health is *Centella asiatica* (CA). CA is a perennial plant that grows in tropical and subtropical countries in moist environments. It is mostly sold under the name Gotu Kola but is also named Indian pennywort, buak bok or kaki kuda [10,11]. Traditionally, CA has been mostly used in the form of tea, but for commercial use it is available as dried aqueous or ethanol extracts. Several studies have shown that CA can improve cognition and has neuroprotective effects [12], but it can also promote wound healing, and it has beneficial effects on various cancers [13,14,15,16]. Although it has been used traditionally to treat anxiety and depression, there are limited scientific and clinical studies addressing its efficacy in depression. One small clinical trial showed that CA reduced anxiety and depression without any side effects in humans [17] but more studies are needed to define the role of CA in anxiety and depression and to identify possible active compounds and mechanisms that mediate its effects. 

*Drosophila melanogaster* flies have long been used as a model to investigate biological processes, including complex behaviors [18,19]. *Drosophila* has also served as a relatively easy system to test drugs and other compounds, including adaptogens used in traditional Asian medicine [20,21,22,23]. We have recently established a model to induce a depression-like state in *Drosophila* by exposing them to chronic, uncontrollable stress [24,25]. Similar approaches have been shown to induce depression in mammalian models and humans [26,27]. We and others also showed that depression can be influenced by modulating serotonin levels and that feeding flies the SSRI fluoxetine, which is widely used in patients, ameliorates the depression-like state [24,28]. 

In this study, we used our *Drosophila* depression model to investigate whether CA can provide resilience against stress-induced behavioral changes. Furthermore, we tested various substances found in CA to identify active compounds that might mediate this effect. Lastly, we initiated experiments to determine molecular targets that mediate its antidepressant properties.

## 2. Materials and Methods

### 2.1. Fly Stock

*D. melanogaster* wild-type Canton S (CS) flies were originally provided by M. Heisenberg, University of Würzburg, Germany. The UAS-Calcineurin A1 RNAi line (*y*^1^ *v*^1^; P{TRiP.JF01871}attP2) and the c739-GAL4 line (*y*^1^ *w*^67*c*23^; P{GawB}*Hr39*^c739^) were obtained from the Bloomington stock center (#25850 and #7362, respectively). The stocks were maintained on standard *Drosophila* food at 25 °C, 60% humidity in a 14 h/10 h light and dark cycle.

### 2.2. Raw Centella asiatica Plant Materials and Pure Compounds

*Centella asiatica* dried aerial parts were purchased through Oregon’s Wild Harvest (Redmond, OR; Batch number X20090016) from an Indian supplier (Organic India). Voucher samples were stored at the Oregon State University (OSU) herbarium (Voucher number OSC-V265416) and in our laboratory at Oregon Health & Science University (OHSU; Voucher number BEN-CA-6). Purified reference *Centella asiatica* triterpenes (TTs; listed in Table 1) and caffeoylquinic acids (CQAs; listed in Table 1) were purchased from Chemfaces (Wuhan, Hubei, Peoples Republic of China). The reference compounds were analyzed and their identities verified by liquid chromatography coupled multiple reaction monitoring mass spectrometry (LC-MRM-MS) and proton nuclear magnetic resonance (^1^H-NMR) analysis as described by Yang et al. [29]. Briefly, ^1^H NMR experiments were performed on a Bruker 700 MHz spectrometer. Proton chemical shift was reported in ppm (δ). The standard sample was dissolved in neat NMR solvent (MeOD). Acquisition of ^1^H NMR spectra was as follows: 16 scans (NS), 3.12 s acquisition time (AQ), collecting 65 k of time domain (TD) data, and 2 dummy scans (DS). The NMR data were processed using Bruker Topspin (ver. 3.5). LC-MRM-MS was performed on a Waters Xevo TQ-XS mass spectrometer coupled to a Waters Acquity UPLC I-Class system. Separation of the standard compounds was achieved using an Intertsil Phenyl-3 column (2 μm, 2.1 × 100 mm) eluting with a gradient of water and methanol each containing 0.1% formic acid [29].

### 2.3. Preparation of Centella asiatica Water Extract (CAW)

A water extract of CA (CAW) was prepared as described earlier by us [29] at the OSU Pilot Food plant. Briefly, CA dried plant material (4 kg) was boiled with deionized water (50 L) in a large kettle for 90 min, periodically replacing the water lost by evaporation. Once heating ceased, the plant material was allowed to settle as the mixture cooled. While still warm but safe to handle, the upper liquid was filtered through a bag filter (McMaster-Carr #5162K112) to remove plant debris and other insoluble materials. The filtrate was frozen in a blast chiller in multiple aluminum baking trays and stored in a frozen state. Lyophilization was performed in 3 separate batches over a period of 18 months to yield extracts BEN–CAW-7, 8, and 9 with a total weight of 820 g (20.5% of the starting dry plant material). The BEN-CAW-7 batch was used in the present study and will be referred to as CAW.

### 2.4. Analysis of the CAW Extract 

To guide the preparation of caffeoylquinic acid (CQA) and triterpene (TT) mixtures containing equivalent levels of CQAs and TTs as in CAW, liquid chromatography coupled to multiple reaction monitoring mass spectrometry (LC-MRM-MS) was applied to analyze and quantify their levels in CAW as described by Yang et al. [29]. To determine the quantity of phytochemical markers in CAW, lyophilized CAW powder (10 mg) was reconstituted in 70% methanol (1 mL) containing 0.1% formic acid and an internal standard digoxin-d3 (1 µg/mL). The re-suspended samples were vortexed (30 s) and sonicated at room temperature (30 min). After centrifugation of the samples (14,000× g, 10 min), the supernatant was analyzed by LC-MRM-MS. The presence of twelve compounds in Centella asiatica water extract was confirmed by comparing retention times with the tolerance limit of deviation (less than 0.05 min), mass spectral fragmentation patterns, and top 3 most sensitive MRM transitions with the corresponding authenticated standards (as in Section 2.2). The content of the 12 compounds in CAW is given in Table 1. 

### 2.5. Supplementing Drosophila Food

To supplement the food, 10× stock solutions were prepared: CAW was dissolved in water (100 mg/mL), and the CQA and TT mixes as well as the single CQA stock solutions were prepared by dissolving commercially obtained pure compounds (Chromadex) in ethanol at concentrations equivalent to CAW (100 mg/mL) based on Table 1. The 10× stock solutions were mixed into standard *Drosophila* food to give the final (1×, if not noted otherwise) test concentrations. Control diets were prepared by diluting the appropriate solvent (water or ethanol) into standard *Drosophila* food.

### 2.6. Analysis of the Drosophila Food

Analysis of selected *Centella asiatica* phytochemical markers in fly food was carried out using the same LC-MRM-MS method as described in Section 2.4. Vials with control and supplemented food were either frozen (−20 °C) immediately after preparation or were placed in the 25 °C fly room for 7 days prior to freezing. To quantify phytochemical markers in fly food, solvent (70% methanol, 1 mL) containing formic acid (0.1%) and an internal standard digoxin-d_3_ (1 µg/mL) were used to extract phytochemical markers from the fly food (50 mg). Samples were vortexed (30 s) and sonicated at room temperature (30 min) with additional vortexing (30 s) in between. After centrifugation of the samples (14,000× *g*, 10 min), the supernatant was analyzed by LC-MRM-MS. 

### 2.7. Stress Protocol

To obtain flies not older than 24 h, newly eclosed CS flies were collected daily. After 2 to 3 days their wings were shortened to prevent flying in the following assays that depend on walking. Cohorts of 10–20 flies were then aged on food vials with vehicle alone, with supplemental CAW, or with compounds for a total of 10 days, with fresh food being provided at day 5. The stress paradigm was initiated at day 10 with repetitive phases of 300 Hz vibrations with cohorts of 10–20 flies confined to empty, narrow tubes during daytime (usually 8 am to 6 pm), as described earlier [23,24]. The stress application was given for three consecutive days, unless indicated otherwise, with flies being transferred to a fresh food vial with or without the supplement overnight. Non-vibrated control flies were confined to the same empty, narrow plastic tubes and placed next to the vibrating device for the same amount of time as the stressed flies. For prophylactic treatment, flies were provided with standard food with vehicle during the nightly rest periods of the three days of stress application. For continuous treatment, flies were returned to supplemented food during each rest period of the stress protocol. A schematic of the treatment strategy is included in each figure showing stress experiments.

### 2.8. Gap-Climbing Assays 

Flies were tested for their motivation to initiate a climbing attempt at a 4.5 mm wide gap, whereby each fly was allowed ten approaches. Only flies that attempted to climb the gap four or more times in the pre-test (PT) were included in the stress protocol to exclude flies that were injured or otherwise damaged (there was no difference in the number of rejected flies between the controls and treated flies). The post-stress tests (T1) were performed after the three days of stress application, unless indicated otherwise, after a short resting period on standard food after the last stress application. An attempt to climb the gap was defined by the stereotypical leg-over-head behavior [23,24]. 

### 2.9. Stop-for-Sweet (S4S) Assay

Flies were collected and subjected to the same feeding and stress protocol as outlined above. After the final day of stress application, flies were placed in empty vials to deprive them of food for at least 8 h. The S4S paradigm was performed as described in Ries et al. [23,24]. Briefly, individual flies were confined to a 55 × 20 mm^2^ chamber made of a 3 mm thick white foam board. The bottom of the chamber was clear plastic and the chamber was covered with filter paper on which a 5-mm-wide stripe of glycerol (99.5%) had been applied along the midline. To induce locomotion, the flies were shaken to the bottom of the chamber and the chamber turned so that the flies walked at a 90° angle upwards on the filter paper. Stopping at the glycerol stripe and extending the proboscis, or continuing walking over the stripe, was recorded by observation. The fly was then immediately shaken down (in case of stopping also to prevent ingestion) and the assay repeated ten times for each fly.

### 2.10. Sleep Experiments

Sleep patterns were assessed using the *Drosophila* activity monitoring systems (DAMS) as described previously [23,30]. Flies were aged for four weeks on standard food and then divided into two groups; one was treated with CAW for two weeks while the other was kept on standard food with vehicle control. The food was replaced with fresh food with or without CAW once during the 2-week period. Individual flies were then placed individually in glass tubes with standard food placed on one end and the other end sealed with a piece of yarn (approx. 1.5 cm). The tubes were placed in DAM2 monitors (Trikinetics, Waltham, MA, USA) and the activity of the flies recorded once every minute. Data from six full days in light/dark cycles (12 h:12 h LD, day 2–8) were then analyzed whereby a period of 5 min or more with no movement detected was regarded as a sleep bout. The data were analyzed using ClockLab (v.6.1.02 for Windows, Actimetrics, Wilmette, IL, USA). Males and females were analyzed separately.

### 2.11. Statistical Analyses

Statistical analyses for the gap climbing and stop-for-sweet assays were conducted using RStudio. Shapiro–Wilk tests were used to test individual data sets for normal distribution. As the data were non-parametric, a Kruskal–Wallis rank sum test was performed, followed by a pairwise Wilcoxon rank-sum test with built-in Bonferroni–Holm correction. Males and females were analyzed separately. The horizontal bar in the box represents the median, boxes represent the 25% and 75% quartiles, while whiskers represent data points within ±1.5 times the IQR. n.s: not significant; *, *p* < 0.05; **, *p* < 0.01; ***, *p* < 0.001 in all figures showing stress data. The sleep data were analyzed using GraphPad Prism 7. Due to the parametric data obtained for the sleep, a 2-tailed unpaired *t*-test with Welch’s correction was performed to compare treated and untreated flies. Males and females were analyzed separately. **, *p* < 0.01. The statistical analyses are presented as Supplementary Data.

## 3. Results 

### 3.1. CAW Is Stable in Standard Fly Food 

**Figure 1:**
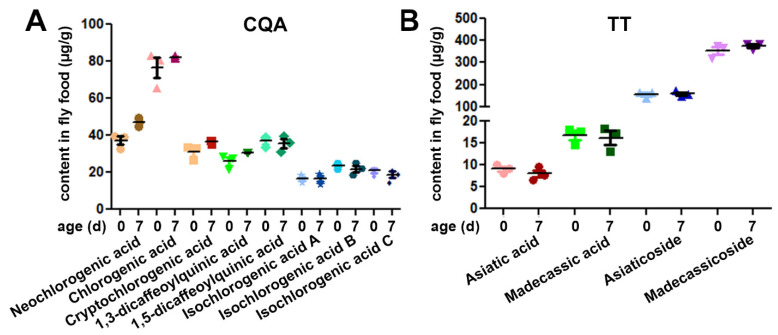
Phytochemical markers for CAW are present and stable in *Drosophila* food containing CAW 10 mg/g. (**A**) CQA levels (µg/g of food) in freshly prepared food (0 d, lighter color) and after 7 days at 25 °C (darker color). (**B**) Measurements of TTs (µg/g of food) in fresh and 7 d old food. n = 3 with triplicate runs. The horizontal bars represent the mean and the SEM is indicated.

*Centella asiatica* has been shown to contain two groups of compounds that have been connected with bioactivity, caffeoylquinic acids (CQAs) and triterpenes (TTs) [12,31]. To confirm that our preparation of the food did not affect CAW composition with respect to the stability of these compounds, we measured the CQAs and TTs as phytochemical markers. As shown in Figure 1, we could detect CQAs (Figure 1A) and TTs (Figure 1B) at levels that were about a tenth of the levels of the 10× stock solution we used to supplement the food (see Table 1). This shows that these compounds are present at the expected levels in the fly food. We also measured these markers in fly food that was left in the fly room for 7 d to determine whether the compounds are stable over the period the food was used. We found similar levels in the 7 d old food compared to the fresh food, showing that the compounds are stable over this time period. 

### 3.2. CAW Ameliorates a Stress-Induced Depressive-like State in Drosophila

**Figure 2:**
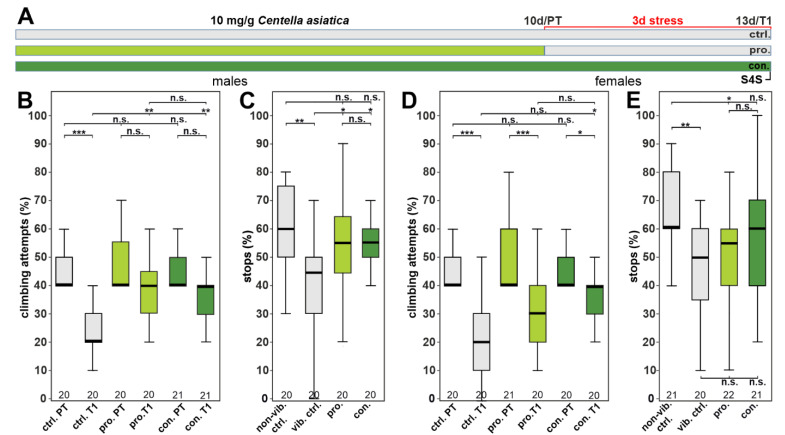
CAW protects males against stress-induced behavioral phenotypes but only partially protects females. (**A**) Schematic of the treatment paradigm with 10 mg/g CAW (treatment schedules are indicated in green). (**B**) Percent of climbing attempts of males before (PT) and after stress (T1) was applied. Prophylactic (pro.) and continuous (con.) treatment with CAW significantly improved climbing. (**C**) Percent of stops males made at the sweet-tasting stripe are increased after both supplementation treatments. (**D**) Percent of climbing attempts of females before and after stress was applied. Only continuous treatment increased climbing significantly but the pre-test motivation level was not reached. (**E**) Percent of stops females made at the sweet tasting stripe after stress were not significantly increased with CAW supplementation. The number of analyzed flies is indicated below the boxes. The horizontal bars in the box plots represent the medians; boxes the 25% and 75% quartiles; whiskers data points within ±1.5 times the interquartile range (IQR). S4S = stop for sweet test; ctrl. = control vib. ctrl = vibrated control; non-vib. ctrl. = non-vibrated control; PT = prestress test; T1 = post-stress test; 10 d = day 10; 13 d = day 13; pro. = prophylactic feeding; con. = continuous feeding. * <0.05; ** <0.01; *** <0.001; n.s. = not significant.

To determine the effects of CAW on a depression-like state (DLS) in our *Drosophila* model, we exposed flies to 10 h phases of vibrational stress for three consecutive days. We then used two assays to measure the effects: One is the gap-climbing assay, which determines the motivation of the flies to climb over an insurmountable gap [23,24]. The other one measures anhedonia by counting how often the flies stop at a sweet-tasting stripe of glycerol, an assay we call the stop-for-sweet (S4S) assay. We also used two treatment strategies during which the flies either obtain supplemented food only during the ten days prior to stress application (prophylactic treatment, pro.) or the supplemented food was also given during the three days of stress induction during the rest period at night (continuous treatment, con., Figure 2A). In previous experiments, testing CAW in a *Drosophila* model of oxidative stress, we found that it had a protective effect at a dose of 10 mg/g of food and we therefore used this dose to test for beneficial effects on depression [32].

To test the efficacy of CAW in the gap-climbing assay, we performed three analyses: First, we determined whether treatment affected the motivation to initiate climbing before being stressed in a pre-test (PT). Second, we compared the performance of stressed, treated flies to stressed, untreated control flies (T1). And lastly, we compared the performance of each experimental group before and after stress was applied (PT versus T1). We included males and females but analyzed them separately to differentiate between possible sexual dimorphisms. When testing males, all groups performed equally well in the pre-test (Figure 2B). However, whereas the untreated flies showed a significant reduction in their motivation to climb the gap after being stressed (T1), both groups receiving CAW, either prophylactically or continuously, were as motivated as before the stress was applied (PT vs. T1 for pro. and con. in Figure 2B, respectively). Furthermore, they were significantly different from the stressed, untreated control flies. A similar result was obtained in the S4S test, with significant improvements after the prophylactic or continuous treatment compared to the stressed, untreated controls (Figure 2C). When testing females, we did find a significant improvement in the gap-climbing test when CAW was given continuously but even then, the flies were less motivated than in the PT (Figure 2D). Female flies that were only treated prophylactically before the stress was applied did not perform better than stressed, untreated controls. In the S4S paradigm (Figure 2E), we did not detect a significant difference between any treatment and the stressed control. However, the flies getting continuous supplementation with CAW were also not significantly different from non-stressed controls. Due to the stronger effects in males, the following studies to identify active compounds in CAW were performed in males only. 

### 3.3. The Protective Effect of CAW Is Mainly Mediated by Mono-CQAs

**Figure 3:**
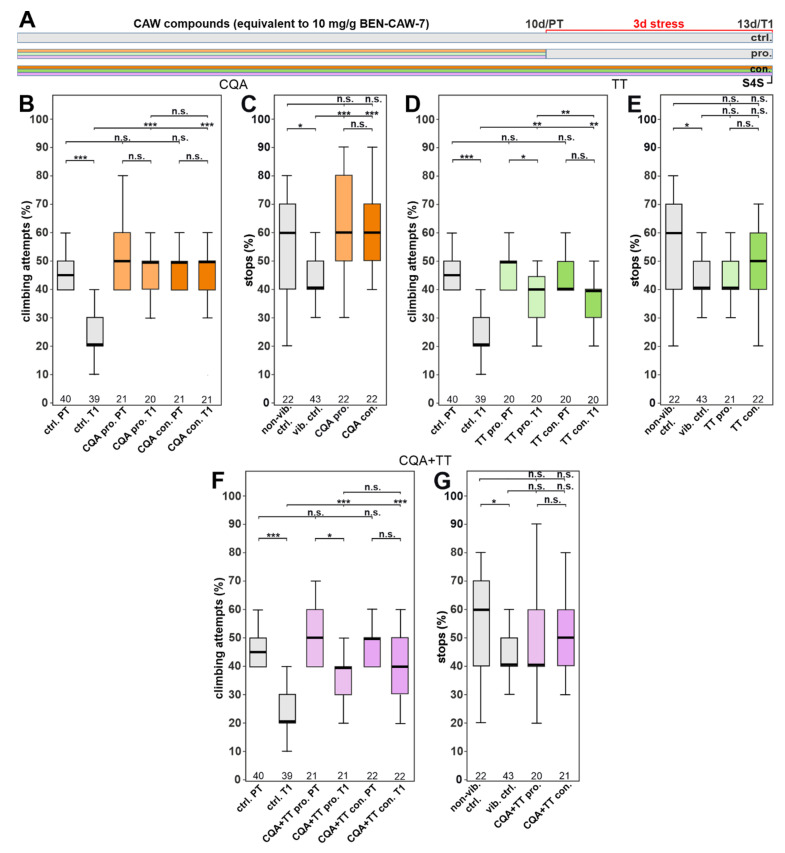
The CQA mix protects against stress-induced behavioral phenotypes, while a TT mix or the combination of CQAs and TTs had only partial effects. (**A**) Schematic of the treatment paradigm with the CQA and TT mixes (at concentrations equivalent to CAW). (**B**) The CQA mix (orange) restored the climbing attempts to pre-test levels after con. and pro. treatment, as well as rescuing the anhedonia-like phenotype (**C**). (**D**) Treatment with the TT mix improved climbing compared to stressed controls after continuous treatment and partially after prophylactic treatment. (**E**) However, it had no effect in the S4S paradigm. (**F**) The combined mix (CQA + TT) could restore climbing to pre-test levels when given continuously but not when given prophylactically; however, it did improve the behavior when compared to stressed controls. (**G**) Similar to the TT mix, the combined mix also had no effect in SFS. All flies tested were males. The number of analyzed flies is given below the boxes. The horizontal bars in the box plots represent the medians; boxes the 25% and 75% quartiles; whiskers data points within ±1.5 times the interquartile range (IQR). S4S = stop for sweet test; ctrl. = control; vib. ctrl = vibrated control; non-vib. ctrl. = non-vibrated control; pro = prophylactic feeding; con = continuous feeding; PT = prestress test; T1 = post stress test; 10 d = day 10; 13 d = day 13; pro. = prophylactic feeding; con. = continuous feeding. * <0.05; ** <0.01; *** <0.001; n.s. = not significant.

As mentioned above, CQAs as well as TTs have been described as active compounds in CAW and we confirmed that they are present and stable in our food preparations. For example, TTs have been shown to protect against neurotoxic insults and stroke-associated neuronal damage, while CQAs can improve age-related cognitive impairment and Aß-induced cytotoxicity [12]. We therefore addressed whether the protective effect of CAW on stress-induced depression-like symptoms is due to either of these compound groups. To supplement the food with CQAs equivalent to the concentration found in CAW the 10× stock solutions were prepared at the concentrations shown in Table 1 and diluted to 1× in the food. We found that this CQA mix restored the stress-induced reduction in gap climbing when given either continuously or prophylactically (Figure 3B). For both treatment conditions, the stressed flies perform as well as before stress was applied. The CQA mix also significantly decreased the anhedonia-like phenotype, tested in the S4S test (Figure 3C). Flies treated prophylactically with the TT mix performed significantly worse in the gap-climbing assay than before stress application after the prophylactic treatment, although they did perform better than the stressed untreated controls (Figure 3D). Continuous treatment did protect against the stress-induced reduction in climbing and restored it to the pre-test level. In the S4S test, prophylactically and continuously TT treated flies showed no improvement in their performance (Figure 3E). Although their performance was similar to untreated stressed flies, the difference to unstressed flies also did not reach significance. Lastly, we tested whether combining the two compound groups resulted in an additive or synergistic effect. The results were very similar to the tests with the TT mix alone as the CQA + TT mixture did restore the gap climbing to pre-stress levels when given continuously and it was partially protective when given prophylactically (Figure 3F). Like the TT mix, the treated flies were not significantly different from the untreated stressed controls in the S4S test (Figure 3G). This shows that there was no positive interaction when combining both compound groups at concentrations of the original CAW extract and that adding the TT mix actually inhibited the stronger protective function of the CQA mix. The stronger effect of CAW compared to CQA + TT also suggests the presence of additional compounds in the entire extract that modulate their interaction.

**Figure 4:**
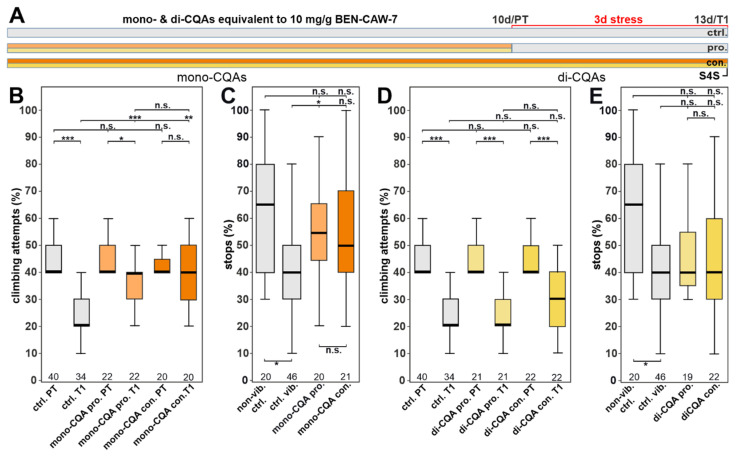
The protective function is mediated by mono-CQAs. (**A**) Schematic of the treatment paradigm with the mono-CQA and di-CQA mixes (at concentrations equivalent to CAW). (**B**) The mix of the three mono-CQAs did restore the climbing attempts to pre-test levels when given continuously, and it also improved the behavior when given prophylactically. (**C**) The percentage of stops was also significantly increased after mono-CQA mix supplementation, but the increase only reached significance after prophylactic treatment. (**D**) In contrast, the di-CQA mix was neither protective in the gap-climbing test, (**E**) nor did it improve their anhedonia-like behavior in the S4S test. Note that the treatment groups in (**E**) are not significantly different from the vibrated controls. Although they are also not significantly different from the non-vibrated controls, the *p*-value for this comparison is 0.03 when comparing directly without Holm correction (see Supplementary Statistical Data). All flies tested were males. The number of analyzed flies is given below the boxes. The horizontal bars in the box plots represent the medians; boxes the 25% and 75% quartiles; whiskers data points within ±1.5 times the interquartile range (IQR). S4S = stop for sweet test; ctrl. = control, vib. ctrl = vibrated control; non-vib. ctrl. = non-vibrated control; pro = prophylactic feeding; con = continuous feeding; PT = prestress test; T1 = post stress test; 10 d = day 10; 13 d = day 13; pro. = prophylactic feeding; con. = continuous feeding. * <0.05; ** <0.01; *** <0.001; n.s. = not significant.

Due to the more marked protective effect of the CQA mix in both gap climbing and S4S compared to the TT mix, we next tested whether a mix of mono-CQAs or di-CQAs provided resilience to stress. Again, we prepared mixes with levels of these compounds equivalent to the levels in CAW (Table 1). As shown in Figure 4B, when given continuously (dark orange) the mono-CQA mix increased the gap-climbing attempts to pre-test levels and was significantly different from the stressed control when given continuously or prophylactically (dark and bright orange, respectively). In the S4S test, the mix of the mono-CQAs did increase the stops after both treatment conditions but it only reached significance after prophylactic treatment (Figure 4C). In contrast, supplementation of the di-CQA mix had no effect, either on gap-climbing behavior (Figure 4D), or on S4S (Figure 4E). 

### 3.4. Single CQA Compounds Do Not Provide Resilience to Stress but a Combination of Chlorogenic Acid and One of Its Isomers Does

**Figure 5:**
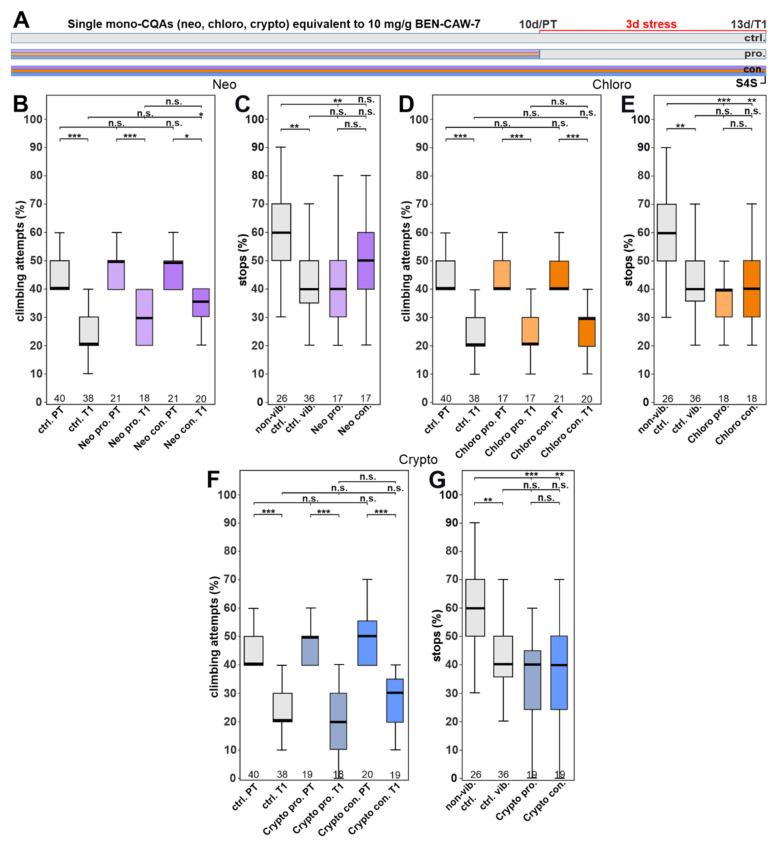
Single mono-CQAs are not effective against the stress-induced behavioral deficits. (**A**) Schematic of the treatment paradigm with the three single chlorogenic acids (at concentrations equivalent to CAW). (**B**) Neochlorogenic acid (Neo) supplementation slightly improved gap climbing when given continuously, but it did not increase the stops for a sweet-tasting stripe (**C**). (**D**,**E**) Chlorogenic acid (Chloro) supplementation did not have an effect in either assay. (**F**,**G**) Cryptochlorogenic acid (Crypto) also did not have a protective effect in either assay. All tested flies were males. The number of analyzed flies is given below the boxes. The horizontal bars in the box plots represent the medians; boxes the 25% and 75% quartiles; whiskers data points within ±1.5 times the interquartile range (IQR). S4S = stop for sweet test; ctrl. = control; vib. ctrl = vibrated control; non-vib. ctrl. = non-vibrated control; pro = prophylactic feeding; con = continuous feeding, PT = prestress test; T1 = post stress test; 10 d = day 10; 13 d = day 13; pro. = prophylactic feeding; con. = continuous feeding. * <0.05; ** <0.01; *** <0.001; n.s. = not significant.

Next, we tested whether supplementation of any of the three mono-CQAs alone is sufficient to protect against stress. We therefore supplemented the food with either chlorogenic acid (Chloro), or its isomers neochlorogenic acid (Neo) and cryptochlorogenic acid (Crypto) at concentrations equivalent to the levels determined in CAW (Table 1). However, we only found a partial increase in performance in gap climbing when Neo was given continuously (Figure 5B; ctrl T1 vs. con. T1), although the flies still attempted to climb significantly less than in the pre-test. We did not detect any improvement in the anhedonia test with any of the individual mono-CQAs (Figure 5C,E,G).

**Figure 6:**
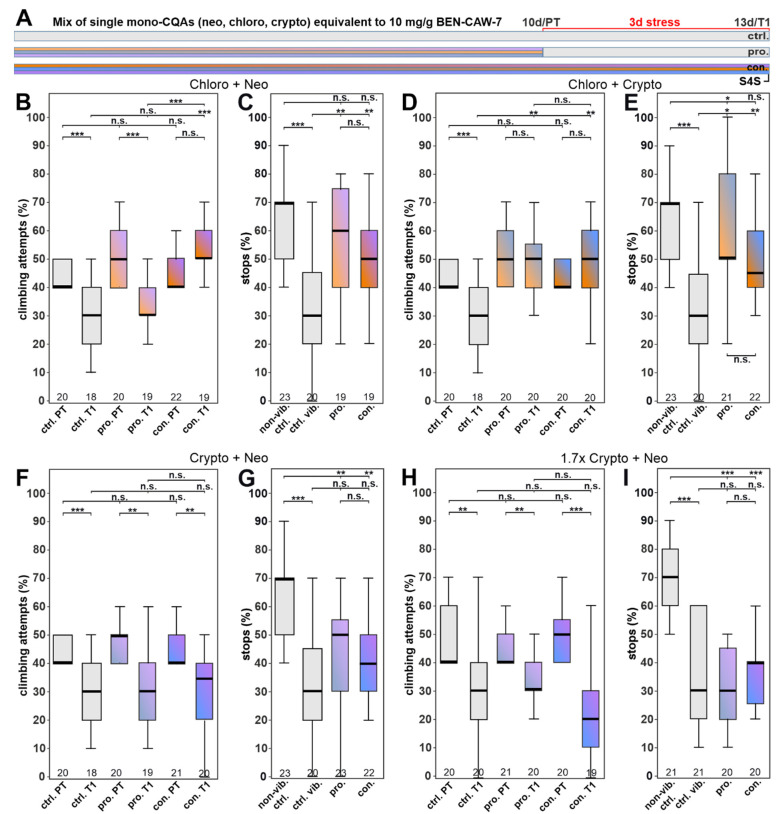
Chlorogenic acid combined with one of its isomers is protective. (**A**) Schematic of the treatment paradigm of combined mono-CQAs (at concentrations equivalent to their levels in CAW). (**B**) Chloro and Neo combined improved gap climbing when given continuously. (**C**) They also increased the stops for a sweet taste after both types of treatment. (**D**,**E**) Chloro in combination with Crypto improved both behaviors, either given prophylactically or continuously. (**F**,**G**) Crypto and Neo combined did not have a protective effect in either assay. Increasing the levels of Crypto and Neo to reach a concentration of mono-CQAs as in the combination with Chloro also had no protective effect, neither in gap climbing (**H**) nor in S4S (**I**). All tested flies were males. The number of analyzed flies is given below the boxes. The horizontal bars in the box plots represent the medians; boxes the 25% and 75% quartiles; whiskers data points within ±1.5 times the interquartile range (IQR). S4S = stop for sweet test; ctrl. = control; vib. ctrl = vibrated control; non-vib. ctrl. = non-vibrated control; pro = prophylactic feeding: con = continuous feeding; PT = prestress test; T1 = post stress test; 10 d = day 10; 13 d = day 13; pro. = prophylactic feeding; con. = continuous feeding. * <0.05; ** <0.01; *** <0.001; n.s. = not significant.

As shown in Figure 4, the mix of the three mono-CQAs did provide resilience but none of the single mono-CQAs did (Figure 5). To address whether the protective function requires all three isomers or whether a combination of two of them is sufficient, we tested pairs of mono-CQAs. As shown in Figure 6B, providing Chloro and Neo did improve the gap climbing when given continuously but not when given prophylactically. Both types of treatment with this combination did improve anhedonia tested in the S4S test (Figure 6C). Combining Chloro and Crypto had a stronger effect, increasing gap-climbing attempts and S4S in both treatment strategies, prophylactically and continuously (Figure 6D,E). In contrast, supplementation with Crypto and Neo had no protective effect in either behavioral test, either when given prophylactically, or continuously (Figure 6F,G). This shows that not all three mono-CQAs are required for the protective function and that a combination of Chloro with one of its isomers is sufficient to provide resilience. However, Chloro is the most abundant mono-CQA in CAW (Table 1) and therefore the overall levels of mono-CQAs are higher in the combinations containing Chloro (1.048 mg/mL and 1.089 mg/mL, respectively in the stock solution). To test whether the increased levels of mono-CQAs in the combinations with Chloro are protective without including Chloro, we increased the concentration of Crypto and Neo 1.7-fold. This results in similar levels (1.084 mg/mL) as in the combinations with Chloro. As shown in Figure 6H,I, this was also not protective in any of the assays, supporting the requirement of a synergistic interaction between Chloro and its isomers. 

### 3.5. Increasing the Concentration of Chlorogenic Acid Can Overcome the Need for Synergistic Isomers 

**Figure 7:**
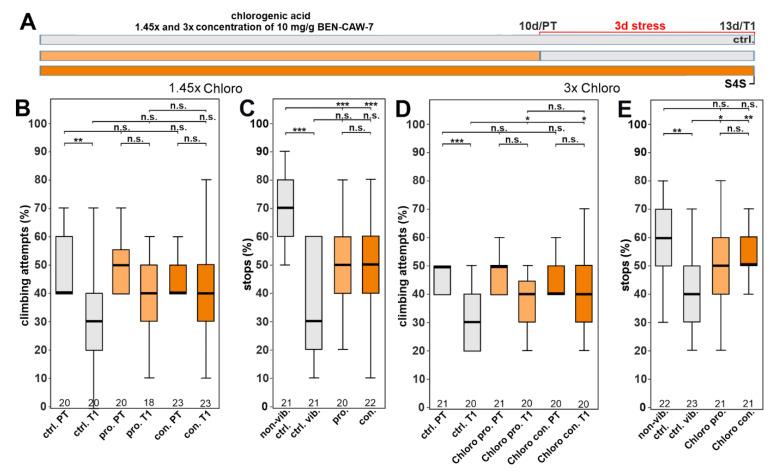
Increasing the levels of chlorogenic acid is sufficient to provide resilience. (**A**) Schematic of the treatment paradigm using increased levels of chlorogenic acid. (**B**,**C**) Increasing Chloro 1.45 fold improved the performance in gap climbing or stopping for a sweet taste but this did not reach significance when compared to untreated controls. (**D**,**E**) Increasing the concentration of Chloro 3 fold protected against both stress-induced behavioral deficits and the stressed flies performed as well as the unstressed flies. All tested flies were males. The number of analyzed flies is given below the boxes. The horizontal bars in the box plots represent the medians; boxes the 25% and 75% quartiles; whiskers data points within ±1.5 times the interquartile range (IQR). S4S = stop for sweet test; ctrl. = control; vib. ctrl = vibrated control; non-vib. ctrl. = non-vibrated control; pro = prophylactic feeding; con = continuous feeding; PT = prestress test; T1 = post stress test; 10 d = day 10; 13 d = day 13; pro. = prophylactic feeding; con. = continuous feeding. * <0.05; ** <0.01; *** <0.001; n.s. = not significant.

The resilience-inducing effect of combining Chloro plus Neo or Crypto, but not Crypto and Neo, suggested that Chloro is essential for the protective function. However, Figure 5D,E shows that Chloro alone at the concentration equivalent to its content in CAW is not protective. To test whether one of the other isomers is required or whether increasing Chloro concentration can overcome the need of the other isomers, we increased the levels of Chloro 1.45-fold (Figure 7A). This resulted in a comparable concentration as in the combination of Chloro with one of its isomers (1.087 mg/mL in the stock solution). Although this did not provide significant protection when compared to the untreated controls, gap climbing was increased to a percentage that was also not significantly different to the pre-test (Figure 7B). Similarly, the treated flies stopped more often for the sweet stripe than untreated ones but again this did not reach significance (Figure 7C). Due to having some effect, we also tested whether further increasing the levels of Chloro would be sufficient to provide resilience. As shown in Figure 7D,E, a 3-fold increase (2.25 mg/mL in the stock solution) did significantly increase the performance in both behavioral assays. Furthermore, it did so when given prophylactically or continuously. We also tested effects of an increase in Crypto and Neo, which did not provide any protection (Supplementary Figure S1). 

### 3.6. CAW Does Not Improve Sleep

**Figure 8:**
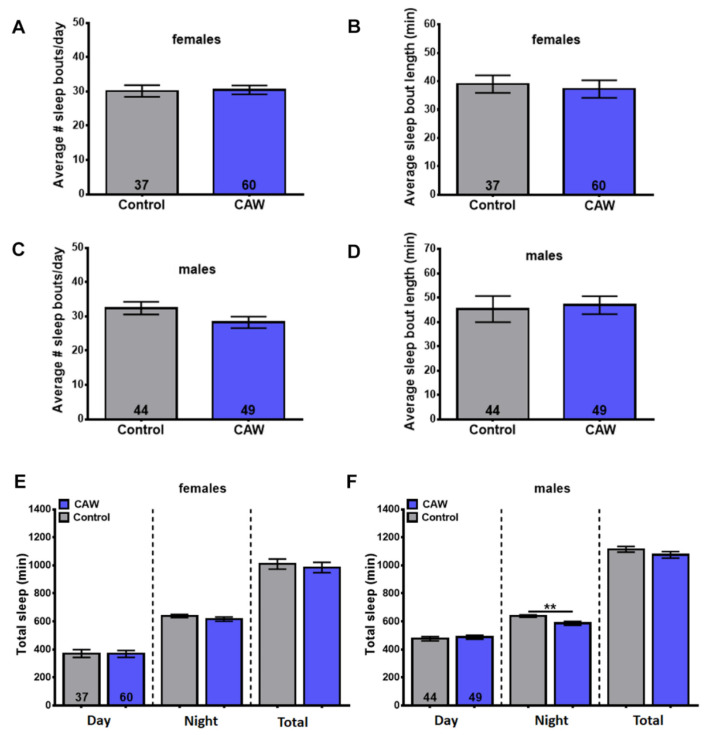
CAW supplementation does not improve sleep. Treating four week old female or male flies for two weeks with 10 mg/g CAW did not have an effect on sleep bout number (**A**,**B**) or sleep bout length (**C**,**D**). It also had no effect on the time female flies spent asleep during the day, night, or in total during a 24 h day (**E**). CAW-treated males showed no significant difference in daytime or total time asleep but nighttime sleep was significantly reduced (**F**). The number of analyzed flies is indicated in the bars. ** <0.01.

Depression is often accompanied by sleep disruptions. Although originally considered to be a consequence, sleep problems have now also been shown to be a risk factor for the development of depression [33,34]. Depression is especially prevalent in the elderly and so are sleep problems [35,36]. Promoting sleep in patients with major depressive disorder improved their depression symptoms and reduced relapses into depression when combined with an antidepressant compared to giving the antidepressant alone [37]. We therefore tested whether CAW has an effect on sleep using aged flies that, as with humans, show increased sleep fragmentation with age [23,38]. The flies were aged on standard food for four weeks, followed by two weeks on CAW or vehicle supplemented food before being analyzed for their sleep pattern. As we previously showed [23], vehicle treated six-week-old flies show sleep fragmentation, as detectable by an increased number of sleep bouts while their duration decreased when compared to younger flies. Measuring sleep bout number and length in CAW-treated flies and untreated controls did not reveal any differences, either in females (Figure 8A,B) or in males (Figure 8C,D). We did also not detect any significant changes in daytime, nighttime, or total time spent asleep in females (Figure 8E), and in males, nighttime sleep was even reduced compared to controls (Figure 8F). This shows that at doses where CAW was active in the other bioassays, it did not improve sleep fragmentation or increase nighttime sleep. 

### 3.7. Calcineurin Depletion Prevents the Protective Function of Chlorogenic Acid 

**Figure 9:**
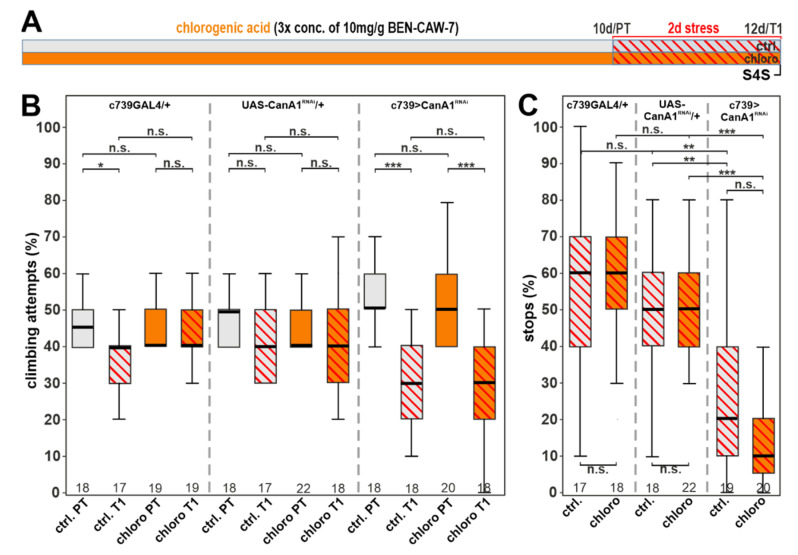
Calcineurin knockdown in the α- and β-lobes of the mushroom body prevents resilience-inducing effects of chlorogenic acid. (**A**) Schematic of the treatment paradigm using chlorogenic acid at a threefold concentration compared to its level in CAW. (**B**) Limiting the stress protocol to two days shows that the knockdown CanA1 flies are more susceptible to vibrational stress in the climbing paradigm (right panel, grey with red stripes) compared to the UAS-CanA1/+ control (middle panel, grey with red stripes) and the c739-GAL4/+ control (left panel, grey with red stripes). When supplementing c739-GAL4 > UAS-CanA1 flies with chlorogenic acid (orange), its resilience-inducing properties are lost compared to the wild-type genetic controls (see Figure 7D). (**C**) This susceptibility and loss of chlorogenic acid’s protective effects are also seen when comparing c739-GAL4>CanA1 knockdown flies in the SFS paradigm (right panel). In contrast, their genetic controls performed normal (left and middle panel, grey). PT = prestress test; T1 = post stress test; 10 d = day 10; 12 d = day 12; pro. = prophylactic feeding; con. = continuous feeding. ** <0.01; *** <0.001; n.s. = not significant.

Lastly, we aimed to identify possible molecular targets that mediate chlorogenic acid’s protective function on depression-induced behavioral changes. One of the possible effectors could be calcineurin, which has been described to be activated by chlorogenic acid [39,40,41]. Calcineurin is a calcium/calmodulin-dependent serine-threonine phosphatase that consists of a catalytic A subunit (CanA) and a regulatory B subunit (CanB) that has binding sites for calcium and calmodulin [42]. To address a possible role of calcineurin in our model, we used a knock-down approach based on the GAL4/UAS system [43]. An RNAi construct against CanA1 under the control of the UAS sequence was activated by crossing the flies to a line containing the c739-GAL4 promoter construct. c739-GAL4 is expressed in the α- and β-lobes of the mushroom body [44], a brain region we have previously shown to be involved in developing a depression-like state in *Drosophila* [24]. When we tested the CanA1 knockdown (c739>CanA1 RNAi) in the stress paradigm, we found that the flies were more susceptible and already showed a significantly reduced performance in gap climbing and S4S after only two days of stress (ctrl. in Figure 9B,C, last panel). In contrast, two days of stress application either had no effect or only a mild effect in flies that expressed only the promoter construct or only the RNAi construct (left and middle panel in Figure 9B,C). This supports our hypothesis that calcineurin does play a role in the depression-like state in our model. To determine whether chlorogenic acid supplementation fails to improve the behavior of CanA1 knock-down flies, we treated them with the three-fold increased concentration of chlorogenic acid that resulted in robust protective effects (as shown in Figure 7D,E). As shown in the right panel in Figure 9B and 9C, the knockdown of CanA1 prevented the protective effect of chlorogenic acid (orange) and the treated, stressed flies performed as badly as the untreated ones (grey). 

## 4. Discussion

CA has been an important medicinal herb in traditional medicine used to improve cognition and memory but also to reduce anxiety and stress [12,45]. Although clinical trials that studied its effect on anxiety and depression have been very limited [17], antidepressant properties have been suggested by some studies in rodents that showed improvement in stress-induced behavior in rats and mice [46,47]. To obtain more insights into the effects of CA on providing resilience to chronic stress and to identify active compounds that mediate these effects, we used a well-established *Drosophila* model. In this model, stress is induced by phases of repetitive 300 Hz vibrations over three consecutive days with rest periods during the night [23,24]. This reduces voluntary behavioral activity, as shown by the gap-climbing assay, and the motivation to stop at a strip with glycerol (stop-for-sweet) which can be compared to anhedonia described in patients with major depressive disorder [48,49]. The CAW water extract significantly improved both behaviors although the effects were much better in males than females. In males, both prophylactic feeding and continuous feeding restored the behavior to the levels before the stress was applied. That CAW is also protective when given prophylactically and not during the stress paradigm itself strongly suggests that it promotes resilience to the stress in contrast to interfering with the mechanisms generating the stress. In contrast to males, the effects of CAW in females were weaker and did not restore pre-stress levels under any condition. We currently do not know why females are less protected and this issue requires further investigation. 

Due to the highly significant protection seen with CAW in males, we used males to address our aim to identify active compounds that mediate this effect. Major compounds present in CA are triterpenes (TTs) and caffeoylquinic acids (CQAs). Concentrations of individual compounds belonging to these two groups were measured in CAW as well as in the fly food. This confirmed that the method of preparation of the food did not degrade or otherwise negatively affect these compounds and that they are stable over the 7-day time period the food was given to the flies. We then tested a mix of eight CQAs, as well as four TTs at levels equivalent to CAW. To our knowledge, CQA and TT mixes have not previously been tested in other depression models. However, Wang and colleagues described that asiaticoside, one of the more abundant TTs, had antidepressant properties in a mild stress model in mice, observing a reduction in the changes in mobility in the forced swim test and adecrease in sucrose consumption [50]. Using the TT mix, we did find a protective effect on the gap climbing but not on the anhedonia phenotype. In contrast, the CQA mix was as effective as CAW and restored both behaviors to pre-stress levels. Combining both groups of compounds surprisingly reduced the positive effects of the CQA mix and although the gap climbing was still improved it was less so when given prophylactically. Furthermore, the performance in the SFS test was not improved by either feeding paradigm using both compound groups. In general, the effects of the combined mixes were very similar to the effects of the TT mix alone. This suggests that other compounds in CAW, which does contain both compound classes, negate the negative effect of the TTs on the CQAs. 

To further narrow down the active compounds, we tested mixes of mono-CQAs and di-CQAs. The latter have been found to be protective against the toxicity of Amyloid-ß in SH-SY5Y cells [51,52] and they also protected cultured hippocampal neurons from dendritic atrophy caused by stress-hormone treatment [53]. However, the di-CQA mix had no effect in our depression model in contrast to the mono-CQA mix, which improved gap climbing and partially improved S4S. Consequently, we tested the three mono-CQAs, Chloro and its isomers Neo and Crypto but none of them were protective when given at the concentration equivalent to CAW. However, combining Chloro with one of its isomers was protective, suggesting either a synergistic effect or a requirement to reach a threshold level of chlorogenic acids to be protective. To address these alternatives, we increased the levels of Crypto plus Neo to the levels reached in the combinations containing Chloro. That this did not provide protection strongly supports a synergistic effect between Chloro and one of its isomers, in contrast to a requirement to reach a threshold level of mono-CQAs. A synergistic effect of Chloro and its isomers is further supported by our finding that increasing Chloro alone to the levels of Chloro plus one of its isomers had no significant effect. However, when we increased Chloro threefold it was as effective as the combination of Chloro with one of its isomers (increasing Neo or Crypto had no effect). This shows that Chloro alone can provide resilience against stress-induced behavioral deficits, although only when given at higher levels than when in combination with its isomers or in CAW. 

Chlorogenic acid is a common polyphenolic compound that is found in several medicinal plants, in fruits, and in coffee beans, where it was first isolated [31,54,55]. In fact, coffee has been shown to have antidepressant effects and though this has been attributed to caffeine, decaffeinated coffee rich in chlorogenic acid can also improve mood [56,57,58]. The antidepressant potential of chlorogenic acid is also supported by experiments in rats and mice, showing that chlorogenic acid or chlorogenic acid-rich plant extracts had anxiolytic and antidepressant effects [59,60,61,62,63]. Due to these findings and our results, we initiated studies to identify a mechanism of how chlorogenic acid may lead to resilience against stress. A possible molecular target was calcineurin which can be bound and activated by chlorogenic acid [39,40,41]. Furthermore, calcineurin has been connected to resilience to depression, as chronic antidepressant treatment of mice results in an increase in calcineurin levels and mice overexpressing calcineurin respond more efficiently to the SSRI fluoxetine [64]. In addition, they found an increase in GluR1, a subunit of the AMPA glutamate receptor in these mice. GluR1 levels have also been shown to be increased after antidepressant treatment of rats [65,66]. This suggests that increased levels or activity of calcineurin is part of the mechanisms of action of antidepressants and that this results in an increase in glutamate signaling. 

In agreement with the protective role of calcineurin, a decrease in its levels should aggravate depression-like symptoms, which we indeed detect as CanA1 knockdown flies were more sensitive to the stress paradigm. Moreover, chlorogenic acid supplementation failed to be protective in these knockdown flies, strongly suggesting that chlorogenic acid’s protective function, and consequently CA’s protective function, is mediated via activating calcineurin. Furthermore, it shows that a loss of calcineurin in the α- and β-lobes of the mushroom body is sufficient to prevent the protective effect of chlorogenic acid.

However, the downstream effects of this interaction of chlorogenic acid with calcineurin remains to be determined. Calcineurin is a multifunctional phosphatase that plays a role in neuronal development, maintenance, and function [42]. Due to these multiple functions, Calcineurin is expressed in many neurons and it is found in the cell body, axons, dendrites, and at synapses [67]. Being activated by Ca^2+^, it can dephosphorylate Glu1 and the NMDA receptor subunit NR2A, thereby regulating the activity of these receptors and neuronal transmission. Furthermore, it has been shown to localize at the pre- and post-synapse where it is involved in synaptic exocytosis and endocytosis. Due to these functions, it plays a role in long-term potentiation and long-term depression as well as in synaptic plasticity [42]. However, calcineurin can also regulate transcription via dephosphorylating the nuclear factor for activated T-cells (NFAT), allowing it to translocate to the nucleus. NFAT can activate the transcription of a variety of genes, including apoptotic genes, genes involved in neuronal and glial development, and as the name indicates, genes involved in immune responses and inflammation [42,68]. Due to the major role of changes in glutamate signaling and AMPA receptor activity in depression [69,70] and the rise in GluR1 in mice overexpressing calcineurin, we hypothesize that the effects of chlorogenic acid may be mediated by changes in neuronal transmission. However, effects on pathways regulated by calcineurin’s transcriptional activity could also play a role in the protective function of chlorogenic acid and CAW, and future studies are needed to address these downstream effects.

## 5. Conclusions

We showed that a water extract of *Centella asiatica* (CA) can provide resilience against stress-induced depression-associated symptoms in a *Drosophila* model. By testing mixes of known compounds of CA, we identified monocaffeoylquinic acids as the active compounds, whereby a synergy between chlorogenic acid and one of its isomers, either cryptochlorogenic acid or neochlorogenic acid, was found. However, increasing the levels of chlorogenic acid alone can overcome the need for this synergistic effect. Using a genetic knockdown strategy, we found that decreasing calcineurin increased the sensitivity to the stress paradigm, and that chlorogenic acid acts via calcineurin. Calcineurin is involved in a plethora of biological processes, including regulating synaptic transmission, and future studies are needed to identify which pathways downstream of calcineurin mediate the protective effects of chlorogenic acid and CA. Our findings provide a basis for future studies in mammalian models to confirm that CA and chlorogenic acid promote resilience against chronic stress and eventually for clinical trials.

## Figures and Tables

**Table 1:** Content of caffeoylquinic acids (CQAs) and triterpenes (TT) equivalent to BEN-CAW-7 (100 mg/mL), the batch of *Centella asiatica* water extract (CAW) used in the present study. This concentration is 10× the final concentration in *Drosophila* food. The data shown in the table are based on three technical replicates of CAW analysis and the standard deviation is indicated. For chemical structures see Yang et al. [29].

Compound Group and Name	Structural Information	Concentration (mg/mL) Equivalent to CAW 100 mg/mL
**CQAs:**		
Chlorogenic acid	3-caffeoylquinic acid	0.750 ± 0.017
Cryptochlorogenic acid	4-caffeoylquinic acid	0.298 ± 0.004
Neochlorogenic acid	5-caffeoylquinic acid	0.339 ± 0.006
1,3-Dicaffeoylquinic acid	1,3-dicaffeoylquinic acid	0.258 ± 0.011
1,5-Dicaffeoylquinic acid	1,5-dicaffeoylquinic acid	0.389 ± 0.006
Isochlorogenic acid A	3,5-dicaffeoylquinic acid	0.177 ± 0.004
Isochlorogenic acid B	3,4-dicaffeoylquinic acid	0.229 ± 0.006
Isochlorogenic acid C	4,5-dicaffeoylquinic acid	0.195 ± 0.007
**Triterpenes:**		
Asiatic acid	Triterpene aglycone	0.042 ± 0.003
Madecassic acid	Triterpene aglycone	0.075 ± 0.001
Asiaticoside	Asiatic acid glycoside	1.475 ± 0.014
Madecassoside	Madecassic acid glycoside	3.589 ± 0.022

## Data Availability

Not applicable.

## Supplementary Materials

The following supporting information can be downloaded at: https://www.mdpi.com/article/10.3390/nu15184016/s1, Figure S1: Increasing the levels of Cryptochlorogenic acid or Neochlorogenic acid does not provide resilience.
