# Supplementary material for: Chlorogenic Acids, Acting via Calcineurin, Are the Main Compounds in Centella asiatica Extracts That Mediate Resilience to Chronic Stress in Drosophila melanogaster
Source: Nutrients. 2023 Sep 16;15(18):4016. doi: 10.3390/nu15184016 (PMC10537055; doi:10.3390/nu15184016)
Supplement: Supplementary file 1 [file nutrients-15-04016-s001.zip › Holvoet et al Supplementary Figure S1.pdf]

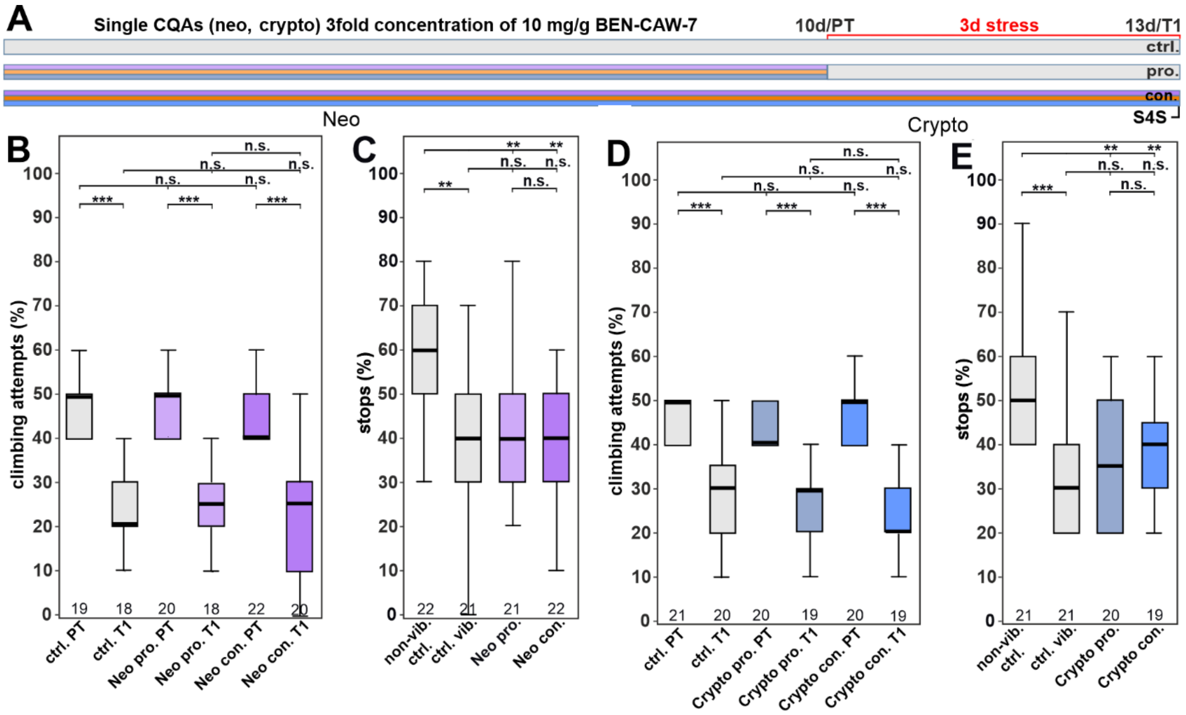

Supplementary Figure S1. Increasing the levels of Cryptochlorogenic acid or Neochloric acid does not provide resilience. (**A**) Schematic of the treatment paradigm using a threefold increase in the concentration of Neo and Crypto when compared to their levels in CAW. (**B, C**) Increased Neo levels does not result in improved gap-climbing or stopping for a sweet taste. (**D, E**) Increasing Crypto did not result in a significant improvement in either assay or treatment paradigm. All tested flies were males. The number of analyzed flies is given below the boxes. The horizontal bars in the box plots represent the medians, boxes the 25 % and 75 % quartiles, whiskers data points within ± 1.5 times the interquartile range (IQR). S4S = stop for sweet test, ctrl. = control, vib. ctrl = vibrated control, non-vib. ctrl. = non-vibrated control, pro = prophylactic feeding, con = continuous feeding, PT = pre-test, T1 = test after being stressed. *<0.05, **<0.01, ***<0.001, n.s. = not significant.
